# Plants as Modulators of Melanogenesis: Role of Extracts, Pure Compounds and Patented Compositions in Therapy of Pigmentation Disorders

**DOI:** 10.3390/ijms232314787

**Published:** 2022-11-26

**Authors:** Anna Merecz-Sadowska, Przemysław Sitarek, Joanna Stelmach, Karolina Zajdel, Ewa Kucharska, Radosław Zajdel

**Affiliations:** 1 Department of Computer Science in Economics, University of Lodz, 90-214 Lodz, Poland; 2 Department of Biology and Pharmaceutical Botany, Medical University of Lodz, 90-151 Lodz, Poland; 3 Department of Medical Informatics and Statistics, Medical University of Lodz, 90-645 Lodz, Poland; 4 Department of Gerontology, Geriatrics and Social Work, Jesuit University Ignatianum, 31-501 Cracow, Poland

**Keywords:** plant, phytochemicals, melanogenesis, signaling pathways, in vivo studies

## Abstract

The kingdom of plants as a “green biofabric” of valuable bioactive molecules has long been used in many ailments. Currently, extracts and pure compounds of plant origin are used to aid in pigmentation skin problems by influencing the process of melanogenesis. Melanin is a very important pigment that protects human skin against ultraviolet radiation and oxidative stress. It is produced by a complex process called melanogenesis. However, disturbances in the melanogenesis mechanism may increase or decrease the level of melanin and generate essential skin problems, such as hyperpigmentation and hypopigmentation. Accordingly, inhibitors or activators of pigment formation are desirable for medical and cosmetic industry. Such properties may be exhibited by molecules of plant origin. Therefore, that literature review presents reports on plant extracts, pure compounds and compositions that may modulate melanin production in living organisms. The potential of plants in the therapy of pigmentation disorders has been highlighted.

## 1. Introduction

The human skin is an important organ in the body and a source of various cells, including melanocytes that residue on the basal layer of epidermis [1]. The main feature of melanocytes is the production of melanin in special organs-melanosomes during a physiological process, called melanogenesis. The quality, quantity and distribution pattern of melanin determines the color of mammalian skin, eyes and hairs. Constitutive pigmentation level of melanin is genetically determined. However, pigment cells have the ability to adopt their melanogenic potential via different internal or external stimuli. The internal stimuli are factors generated inside the organism, whereas the external stimuli are environmental agents including the most potent one, i.e., ultraviolet (UV) radiation [2].

Melanogenesis consists of different stages and involves action of various enzymes. Tyrosinase (TYR) is the key enzyme, which takes part in melanin synthesis by catalyzing hydroxylation of L-tyrosine to 3,4-dihydroxy-phenylalanine (DOPA) and oxidation of DOPA to DOPAquinone. The produced pigment is transferred from melanocytes into keratinocytes. This compound constitutes the first line of defense against UV radiation and provides scavenging of reactive oxygen species (ROS) [3]. However, the disturbance of melanogenesis process may lead to overproduction, deficiency, abnormal transport or transfer of melanin, which in turn may result in pigmentation disorders [4].

Numerous studies showed a beneficial effect of plant extracts or pure compounds in various skin aliments. According to literature, plants provide, e.g., antioxidant, antiaging and anti-inflammatory properties [5,6]. Such an effect is due to the presence of plant secondary metabolites. These molecules, characterized with high structural diversity, belong to different chemical classes, including phenolics, terpenes, alkaloids, saponins, lipids and carbohydrates [7]. Many of them are able to modulate different signaling pathways, including those which regulate the expression and activity of melanogenesis-related proteins. The most crucial signaling pathway is mediated by the melanocortin-1 receptor (MC1R). Other pathways include those mediated by the tyrosine kinase receptor (c-KIT), Frizzled receptor and endothelin B receptor (ETBR). Microphthalmia transcription factor (MITF) is a downstream target, being the main regulator of melanogenesis. MITF propagates melanogenesis by induction of the expression of TYR and other pigmentation genes, including tyrosinase-related proteins, i.e., TYRP1 and TYRP2. Therefore, up-regulation or down-regulation of such signal transduction is one of the most desirable ways in the therapy of skin hyperpigmentation or hypopigmentation [8,9].

Studies focused on the modulatory role of molecules of plant origin on melanogenesis mechanisms, are performed on various model systems, including in vitro and in vivo. The in vitro model comprises studies that examine the influence of phytochemicals on melanocytes in monolayers whereas the in vivo model comprises studies investigating the influence of phytochemicals on living organisms. Numerous approaches are currently available, but each has its own advantages and limitations. Moreover, growing data on possibility of modification of melanogenesis-related pathways by plants is followed by an increasing number of patented compositions.

The aim of the study is to present the role of plants, including extracts, pure compounds and patented compositions as modulators of melanogenesis process in living organisms. Their beneficial effects on skin and applicability in the therapy of pigmentation disorders have been also highlighted.

## 2. Study Design

This literature review includes data published in the years 2012–2022 obtained from NCBI-PubMed, Google Scholar, Scopus, and ScienceDirect databases. The following keywords were selected: plant extract, plant pure compound, melanogenesis, signaling pathways, in vivo study. Studies on plant extracts and pure compounds as well as their impact on the melanogenesis process in in vivo model were explored. Only compounds isolated directly from plants were included. In addition, patented compositions were added. An analysis of cell lines in monocultures was rejected. Items published in languages other than English or containing only an abstract were also excluded. In order to standardize scientific names of plants, the “Medicinal Plant Names Services” (https://mpns.science.kew.org/mpns-portal/searchName?) (accessed on 12 September 2022) was used. PuBChem (https://pubchem.ncbi.nlm.nih.gov) (accessed on 12 September 2022) enabled to obtain IUPAC names of pure compounds.

## 3. Melanocyte Characteristics

Melanocytes are a heterogeneous group of cells found in different locations in a human body. They consist of the central body and dendrites. Melanocytes originate from melanoblasts, neural pluripotent cells of neural crest. Dermal melanocytes derive from several populations of neural crest cells, including cranial, dorsal and ventral trunks. Melanoblasts successively undergo migration, proliferation and differentiation into melanocytes. Mature cells acquire the ability to produce melanin in special organelles, called melanosomes. There are two types of dermal melanocytes. One is present in the basal layer of the epidermis, while the other is found within hair follicles. Melanocytes located in the epidermis account for 5–10% of all cells. They form epidermal melanin units, which are a dendritic junction of one melanocyte with about 30–40 neighboring keratinocytes. Dermal melanocytes transport mature melanosomes into keratinocytes, which is followed by cell death [2,10,11].

Cross-talk between melanocytes and keratinocytes and fibroblasts is demonstrated. Melanocyte biology is controlled through various signaling molecules. Keratinocytes-derived factors, such as basic fibroblast growth factor (bFGF), stem cell factor (SCF), or endothelin 1 (ET-1), initiate melanocyte division. Moreover, keratinocytes upon UV radiation produce the following factors: bFGF, ET-1, interleukin (IL) IL-1α/1β, adrenocorticotropic hormone (ACTH), melanocyte-stimulating hormone (α-MSH), prostaglandin E2 (PGE2), PGF2α, granulocyte-macrophage colony-stimulating factor (GM-CSF), nitric oxide (NO), tumor necrosis factor α (TNF-α), nerve growth factor (NGF), bone morphogenetic protein 4 (BMP-4). They affect proliferation, dendriticity, melanogenesis efficiency and survival of melanocytes. Fibroblast-derived factors, such as bFGF, hepatocyte growth factor (HGF), or endothelin 3 (ET-3), initiate melanocyte division. Fibroblasts also produce stem cell factor (SCF) and neuregulin 1 (NRG1) which determine melanocyte growth, shape and motility. These paracrine regulations play a very important role in maintaining skin homeostasis.

The melanocyte itself also secretes a number of signaling molecules such as pro-inflammatory cytokines IL-1α, IL-2, IL-3, IL-6, IL-10 and TNF-α. Some of these, including L-1, IL-6 and TNF-α inhibit melanogenesis. In contrast, factors secreted by melanocytes that stimulate melanin production are eicosanoids and melanocyte stimulating factor (α-MSH). Other factors produced by melanocytes include transforming growth factor (TGF-β), chemokines, catecholamines, serotonin and NO [2,12,13].

The primary function of melanocyte is melanin production. Melanins are responsible for pigmentation of the skin, as well as hair or eyes. There are two main groups of melanins: eumelanins and foemelanins. Eumelanins give a brownish to dark black color while foemelanins give a yellowish or reddish color. Pigment amount varies depending on the activity of melanosomes, differences in the production and deposition. A higher content of pheomelanin relative to eumelanin is observed for fair skin, while the opposite ratio is observed for dark skin. There are no significant differences in melanocyte density in different racial groups. However, the density is different in a different body region. It is estimated that there are between 900 and 1500 melanocytes per mm^2^ on the back and genital area on average about 1000 melanocytes per mm^2^ [14].

Biochemical pathway leading to synthesis of melanin is called melanogenesis. It is estimated that pigment production is regulated by more than 250 genes that determine melanocyte development, migration, proliferation, differentiation, and survival [15,16]. Pigment cells express among others TYR, TYRP1, TYRP2 and MITF proteins which are closely related to melanogenesis. TYR, being an oxidase located in the membrane of melanosomes, is responsible for converting L-tyrosine, a precursor of melanins to DOPAquinone [17]. Apart from TYR, TYRP-1 and TYRP-2 are other enzymes which are also involved in melanogenesis. These proteins are structurally similar to TYR. TYRP1 is an oxidase involved in the final stage of melanogenesis; it can increase the ratio of eumelanin to pheomelanin and it is also involved in activation and stabilization of TYR by forming a complex with it. TYRP2 is a tautomerase responsible for the conversion of DOPAchrome to carboxylic acid derivatives. In the melanosome, it forms complexes with TYR and TYRP1 [18]. MITF is a transcription factor which recognizes the sequences of TYR, TYRP1 and TYRP2 genes and modulate their transcription. The signaling cascade involving MITF is one of the most important for melanogenesis regulation [19].

Protection against UV radiation is the primary function of melanins. Melanins have both UV absorbing and scaterring properties. Black epidermis transmits only 7.4% of UVB and 17.5% of UVA, whereas 24% of UVB and 55% of UVA pass through white skin. This role is very important due to the fact that UVB radiation induces DNA bases damage, whereas UVA radiation leads to production of ROS as well as single strand breaks or crosslinks between DNA and proteins. Photodamage can cause mutations in critical genes. Therefore, UV radiation is a primary harmful environmental factor, inducing occurrence of skin cancers such as malignant melanoma, squamous cell carcinoma and basal cell carcinoma. In turn, melanosomes transferred from melanocytes to keratinocytes form characteristic perinuclear caps that create a DNA protection shield. In addition to its UV radiation protective role, melanin also exhibits antioxidant activity as well as anti-inflammatory and immunomodulatory properties [20,21,22].

## 4. Plant Extract and Pure Compounds–Biological Properties and Modulatory Effect on Melanocyte

Plants are a rich source of phytocompounds. It is estimated that any single species may contain as many as 5000 metabolites [23]. These are chemically diverse substances that can play many different roles, including determination of plant growth and development, protection against pathogens, and response to environmental stress. The metabolites produced by plants are classified as primary and secondary. The second group includes mainly intermediates or by-products of primary metabolism. Terpenoids, alkaloids, phenylpropanoids, polyketides, quinones and cyanogenic glycosides are formed in the process of carbon and nitrogen metabolism; glucosinolates are formed by sulfur metabolism, while alkylamides are formed by fatty acid metabolism. [24]. Plant secondary metabolites may also positively affect the human body [25]. Some of these substances demonstrate therapeutic effects on cells or tissues and may be useful in alleviating numerous aliments [26]. In addition, their contribution to disease prevention is being considered [27]. A lot of efforts are put to isolate highly desirable molecules and to determine their chemical structure, as well as activity, functions and toxicity [28,29].

Annual production of plants, characterized with medicinal properties, is worth more than $100 billion [27]. Molecules of natural origin may have several properties, including photoprotective [30] as well as anti-inflammatory [9], antioxidant [25] or anticancer [26] via modulation of cellular signaling pathways [31,32,33,34,35,36,37,38].

Plant-derived compounds may counteract the harmful effects of UV exposure on human skin. Phenolic compounds are the most important class of phytochemicals that may be used as sunscreen agents. These molecules equipped with aromatic rings are able to absorb UVA and UVB radiation at wavelengths of 200–400 nm. Two flavonoids, quercitin and rutin at 10% (*w*/*w*) concentrations provide SPF values of about 12 [39]. Moreover, combining 0.1% (*w*/*w*) rutin with 6% benzophenone, a synthetic organic filter, increased the SPF value from about 24 to 33 [40]. Similarly, synergistic effects were observed between 0.1% rutin (*w*/*w*), 1% benzophenone (*w*/*w*) and 3.5% (*w*/*w*) ethylhexyl methoxycinnamate, another synthetic organic filter [41]. Choquenet tested twelve phenolic compounds at various concentrations, including myricetin, apigenin, luteolin, puerarin, baicalin, baicalein, naringenin, hesperidin, hesperetin, diosmin, caffeic acid and chlorogenic acid. Of these, apigenin and chlorogenic acid were found to be the most effective UVB and UVA filters, with SPF values at about 7 and 10, respectively [42]. Similarly, among fifteen tested phenolic compounds at 7% (*w*/*v*) concentrations (resveratrol, quercetin, catechin, kaempferol, piceid, galangin, apigenin, naringenin, chrysin, pinocembrin, ferulic acid, coumaric acid, caffeic acid, caffeic acid phenylethyl ester and dimethyl caffeic acid), the highest SPF value was observed for apigenin, i.e., about 28.8 [43]. In addition, studies show that plant extracts from different plant species have also a photoprotective action, especially plants of the species which stand out large amounts of phenolic compounds [30].

Bioactive compounds are known to improve the anti-inflammatory response via downregulation of different inflammatory pathways, including mitogen-activated protein kinases (MAPKs), nuclear factor kappa-light-chain-enhancer of activated B cells (NF-κB) and activators of transcription (JAK/STAT) pathways. This stimulation contributes to production of various pro-inflammatory mediators, while their inhibition bestows an anti-cancer effect.

Oxidative stress is implicated in the pathogenesis of many chronic diseases, including diabetes, cardiovascular diseases, atherosclerosis, rheumatoid arthritis, chronic inflammation, cancer, aging, and various other neurodegenerative disorders. ROS activates signaling pathways, such as MAPK, NF-κB, nuclear factor erythroid 2–related factor 2 (Nrf2) and various transcription factors, including activator protein 1 (AP-1), tumor protein p53, hypoxia-inducible factor 1-alpha (HIF-1α), peroxisome proliferator-activated receptor gamma (PPARγ), and signal transducer and activator of transcription (STAT-3). Nrf2 pathway upregulates cell defense mechanisms and antioxidant gene expression. This pathway is known to be stimulated by plant-derived chemicals with antioxidant properties, thus providing beneficial effects against ROS-related disease, including cancer.

Phytochemicals also exert a direct detrimental effect on cancer cells by cell cycle arrest via downregulation of (MAPKs) and phosphoinositide 3-kinases (PI3K)/protein kinase B (AKT) pathways [44]. Data indicate that suppression of PI3K/AKT signaling pathway leads to inhibition of proliferation, apoptosis, induction of autophagy of cancer cells [45]. In addition, molecules of natural origin can block cancer cell differentiation and angiogenesis by downregulation of the HIF-1α pathway and vascular endothelial growth factor (VEGF), and influence cancer cell survival by altering p53, death receptor expression and pro-apoptotic:anti-apoptotic protein balance [44].

Secondary metabolites obtained from plants can also modulate signaling pathways involved in the melanogenesis process in melanocyte cells. A lot of studies have been focused on the ability to regulate MC1R, c-KIT, WNT and ETBR pathways.

Induction of G-protein-coupled receptor MC1R is a canonical regulatory mechanism of melanin production by melanocytes. This occurs through its agonist, α-melanocyte-stimulating hormone (α-MSH) bounding. Activation of adenylyl cyclase is followed by an increase in the cyclic adenosine monophosphate (cAMP) level. cAMP is a second messenger produced from ATP by adenyl cyclase that regulates numerous cellular functions, including growth, proliferation, differentiation, migration, as well as genes expression. Protein kinase A (PKA) is one of effector proteins, which gets activated by high cAMP levels. Consequently, PKA activates target proteins including cAMP response element-binding protein (CREB) [46]. PKA is responsible for Ser133 phosphorylation of CREB, which is then followed by CREB-mediated MITF expression. It was also observed that the binding of α-MSH to MC1R contributes to triggering PI3K/AKT signaling pathway. PI3K activity results in recruitment of AKT to the cell membrane, where phosphorylation and activation of AKT occur. A direct target for AKT is, among others, glycogen synthase kinase 3β (GSK3β), which then phosphorylates on Ser298 and activates MITF [47,48,49]. Data also indicate that AKT can directly phosphorylate MITF on Ser510 [42]. In turn, cAMP levels in the cell can modulate the MAPKs pathway. Increased cAMP amounts induce ERKs and inhibit p38 activation [50]. Active ERK phosphorylates MITF on Ser73 and triggers proteasomal degradation mediated by ubiquitin [51]. In contrast, active p38 causes CREB phosphorylation and MITF activation [52]. Similarly, PI3K/AKT and MAPKs signals also regulate numerous cellular functions, including proliferation, differentiation and migration [53,54].

MITF can be also regulated via c-KIT receptor and its ligand stem cell factor (SCF) junction, which is dependent on PI3K/AKT and p38 signaling [55]. Data indicate that induction of c-KIT constitutively stimulates PI3K by its phosphorylation, as well as activate Src-homology 2 domain-containing proteins, of which the Src family kinases promote p38 action [56].

WNT signaling is initiated by binding of Wnt ligands to Frizzled receptor-related protein receptor complexes. In the absence of Wnt ligands, β-catenin phosphorylation by GSK3β results in its degradation by a protein complex consisting of Dishevelled, GSK3β, axin or conductin, and the adenomatous polyposis coli tumor suppressor protein. In the presence of Wnt ligand, β-catenin phosphorylation is blocked. This provides β-catenin stabilization followed by nuclear translocation, where it can interact with TCF/LEF transcription factor and regulate the expression of downstream genes. MITF is one of positively regulated genes targeted by WNT signaling [57,58].

ETBR is also a G-protein coupled receptor. Its activation by ligand–endothelin 3 (ET-3) results in elevated phosphorylation of ERK, CREB and MITF. ETBR acts synergistically with MC1R. The action is followed by MITF activation and increased expression of its target genes, including TYR, TYRP1 and TYRP2. In addition, increased phosphorylation of AKT was also observed [59].

Modulating of MITF activity can affect the efficiency of melanogenesis. In some cases, inhibition of MITF activity leads to reduced melanin production, while in some others, stimulation of MITF activity results in increased melanin production. Both the effects may be achieved through implementation of plant-derived compounds [60]. Therefore, these molecules may exert a beneficial therapeutic effect on skin of patients with hyperpigmentation or hypopigmentation, including postinflammatory hyperpigmentation, solar lentigines, melasma, café au lait macules, ephelides (freckles) as well as postinflammatory hypopigmentation, vitiligo, pityriasis alba, tinea versicolor [61,62,63,64,65].

## 5. The Effect of Plant Extracts or Pure Compounds on Melanin Content in the *Danio rerio* Model

*Danio rerio* (zebrafish) is a popular model for biomedical research. It is characterized with fully sequenced genome, being approximately in 70% orthologues to the human genome, easy genetic manipulation, rapid development, high fecundity, external fertilization, transparent embryo and presence of main organs necessary for metabolism. Moreover, that small freshwater fish is also easy to breed and maintain [66].

The zebrafish is also highly desirable in dermatological studies due to the fact that components of the epidermis and dermis get formed just 1 day post-fertilization (dpf), whereas skin structure is fully developed 6 dpf. As a consequence, after 6 dpf zebrafish skin is composed of multilayer epidermis and collagenous stroma with well-demarcated keratinocytes and numerous fibroblastic cells. Besides, presence of a pigment cell system is a crucial feature of zebrafish skin [67,68]. Interestingly, zebrafish skin is similar to human skin and consists of the epidermis, dermis and hypodermis. Unlike human skin, it does not have a keratinized outer layer and mammalian appendages, including sebaceous glands and hair follicles. However, studies on that non-mammalian vertebrate model constitute a fundamental part of skin-related research [69].

The zebrafish model is getting more and more applicable in exploration of pigment disorders due to having a conserved melanogenesis pathway and visible melanin development in melanophores from 1 dpf. The zebrafish pigment cells are derived from neural crest stem cells. They generate melanophores. Melanocytes and melanophores look alike similar. Melanophores are located in the epidermis and hypodermis and they do not transfer melanin, while melanocytes residue in the epidermis and transfer melanin to keratinocytes. However, the biology of melanophores is similar to that of melanocytes. In that model organism, numerous genes which control pigment production and function of melanocytes, such as MITF, TYR and TYRP1, are conserved and similar to those observed in a human body. In addition, regulation of MITF activity by signaling pathways, including MC1R, WNT, c-KIT and ETBR, has been found in both human and zebrafish pigment cells [70]. Studies on melanogenic potential of plant extracts and pure compounds of the zebrafish embryos are presented in Table 1 and Table 2. According to that studies, analyzed extracts and pure compounds were mostly noncytotoxic towards zebrafish embryos in the range of tested concentration.

## 6. The Effect of Plant Extracts and Pure Compounds on Melanin Content in the Rodent Model

The rodent model allows both to evaluate the impact of molecules on the whole organ, i.e., the skin, and to observe interactions of this organ with other body organs, which makes this model advantageous. On the other hand, results of the rodent model obtained in preclinical studies are sometimes impossible to translate into clinical practice. Of all rodent models, the mouse model is most commonly used. Mice are easy to breed and maintain. They are genetically modifiable, have a short lifespan, and their skin properties are similar to those observed in human skin. However, there are some structural and molecular differences. The most obvious difference is the fact that rodent skin is more permeable than human skin, which is contributed by fur which covers most of its body. Besides, it is also thinner than human skin. Body areas not covered by fur, i.e., the ears and tail, have thicker epidermis, like in humans. In addition, the skin of rodents contains muscle tissue, which is not found in human skin and epidermal regeneration takes 8–10 days, whereas this process in humans takes 26–28 days. Moreover, a different distribution of melanocytes is observed in mice. They are found mainly in hair follicles, while in humans, they are present in both hair follicles and the basal layer of the epidermis [103].

Studies conducted on the rodent model and investigating melanogenic potential of plant extracts and single compounds which are administered topically are available in the literature. The experiment lasts on average from a few days to several months. The shortest-lasting research was conducted on UVB-irradiated HRM-2 hairless mice exhibited to *Pueraria thunbergiana* in the form of cream containing 1 or 3% of the extract on the dorsal skin. Findings show great efficacy of the extract on pigmentation and melanogenesis induction after 3 days of exposition [104]. Jegal et al. tested *Juniperus communis* extracts at concentration of 5 to 50 μg/mL for 11 days as well as their main constituent, i.e., hypolaetin-7-O-β-D-xylopyranoside at a concentration of 50 μM on UVB-irradiated HRM-2 hairless mice. Solution at 200 µL volume was applied to a dorsal skin site 3 × 3 cm. Histological findings of skin samples indicate a reduction in melanin deposition after extract treatment and a reduced number of melanocytes after exposition to pure compounds [105]. *Nelumbo nucifera* extract, used at 1% or 2% concentration and administered to UVB-irradiated guinea pigs on the back region for 2 weeks demonstrates similar activity. The main identified constituent was gallic acid [106]. Data show that 200 μL of *Nymphaea nouchali* extract, applied on UVB-irradiated HRM-2 hairless mice at a concentration 100 µg/mL to a 3 cm × 3 cm designated area on the dorsal skin for 4 weeks exhibit antimelanogenic effects [107]. *Medicago sativa* extract used at 10% or 20% concentrations and administered to dorsal region of C57/BL6 mice for 4 weeks exhibit a remarkable skin and hair pigmentation [108]. A study conducted by Choi et al. on UVB-irradiated brown guinea pig skin models revealed that *Cyrtomium fortunei* used at 3% concentration on back area and administered for 6 weeks exhibited depigmenting properties [109]. In addition, UVB-irradiated C57BL/6 mice treated with *Nelumbo nucifera* extract at concentrations of 1.25 mg/cm^2^ and 2.5 mg/cm^2^ for 8 weeks inhibited melanin production in the ear skin sample. The main component of this extract was identified as epigallocatechin [110]. *Pyrostegia venusta* extract used at 10% and administered to dorsal region of Swiss mice mice for 65 days show a increased epidermal melanin level and diminished dermal depigmentation [111]. The longest studies, covering a period of 5 months, were conducted on C57BL/6J Ler-vit/vit mice. *Lespedeza bicolor* extract applied at an amount of 0.2 mL/cm^2^ to those animals significantly increase the level of melanocytes in the skin. The main constituent of extract was identified as quercetin [112]. Application of 0.2 mL/cm^2^ *Rhododendron schlippenbachii* extract for 5 months leads to melanin production [113].

Other research involves oral administration of plant extracts or pure compounds. The experiments lasted from few days to several weeks. The shortest-lasting study was provided by Niu et al. on SD rat serum exposed to *Caesalpinia sappan* extract, applied at a dose of 1.15 g/kg, significantly reduced the content of TYR. Isolated compounds were identified as sappanone B, brazilin, protosappanin A, protosappanin B, caesalpin J [114]. C57BL/6J mice exposed to *Adenostemma lavenia* extract administered at a concentration of 100 mg/kg for 2 weeks show inhibited hair pigmentation. The main constituent was identified as ent-11-hydroxy-15-oxo-kaur-16-en-19-oic acid [115]. Improved depigmentation was observed after UVB-irradiated HRM-2 hairless mice treatment with *Panax ginseng* extract at doses of 150 and 300 mg/kg for 5 weeks [116] as well as 100 and 200 mg/kg of *Zingiber mioga* extract for 6 weeks [117]. Moreover, C57BL/6 mice and brown guinea pigs undergoing hydroquinone and H_2_O_2_ induced hypopigmentation, respectively. Both were then treated with *Vernonia anthelmintica*, administered at concentrations of 61.5–246 mg/kg to the mice and 22–88 mg/kg to the guinea pigs for 30 days. In consequence, the extract darkened the dorsal skin samples and hair both in the mice and the guinea pigs [118].

## 7. The Effect of Plant Extracts or Pure Compounds on Melanin Content in the Human Model

In the human model, analyzing plant extracts were administered primarily topically. The research was mainly focused on an analysis of extracts with whitening properties. The experiments lasted an average several weeks. The shortest-lasting studies, i.e., covering the period of 4 weeks, involved application of *Perilla frutescens* extract at a concentration of 0.1% in 30 patients to the forearms [119] and *Salacca edulis*, rich in flavonoids extract, applied at 3% concentration to the forearm of 17 participants [120]. Other studies included exposure of 24 volunteers to 1% *Etlingera elatior* extract [121] and 30 subjects to *Piper betle* extract applied at a dose of 5 mg/cm^2^ [122] to the forearm. Another studies involved 30 volunteers that tested 0.2 or 0.3 g of *Perilla frutescens* [123] and phenolic-rich *Punica granatum* extracts [124], applied to the face. *Panax ginseng* extract was applied as 3% cream by 10 volunteers onto foreheads and cheeks [116]. *Gnetum gnemon* extract at 3.5% concentration was applied to upper arms of 38 patients [125]. A study lasting 6 weeks evaluated the effect of *Rosa gallica* 0.05%, extract on facial skin of 10 participants [126] and *Chrysanthemum indicum* 0.5% extract on facial skin of 30 patients. It was revealed, that luteolin and acacetin-7-O-rutinoside are major flavonoid compounds in that extract [127]. For 7 weeks, 0.05 or 0.1% *Litchi chinensis* extracts were tested on 29 volunteers on the forearm [128]. Some other studies covered a period of 8 weeks. The 3% *Crocus sativus* extract was applied to cheeks and forearms by 10 volunteers [129] and 68.6% *Aster yomena* extract was applied to the face by 22 participants [130]. The effect on humans facial skin of a single compound isolated from *Pterocarpus marsupium*, called pterostilbene and administered at 0.4% concentration was evaluated in 38 participants for 8 weeks [131]. Another study took 12 weeks. A study conducted by Ali et al. evaluated phenolic-rich *Acacia nilotica* 3% extract [132], while that carried out by Khan et al. evaluated both *Hippophae rhamnoides* and *Cassia fistula* extracts applied at a concentration of 500 mg to cheeks of 11 and 25 volunteers, respectively [133].

On the other hand, oinment with *Piper nigrum* extract as well as its constituent–a alkaloid piperine at concentration of 5 mg/mL and 2 mg/mL, respectively promote melanocyte activity. Pigmentation was achieved in all the treated areas of 3 vitiligo patients during 12 weeks [134]. Similar effects was observed for 20 patients treated for 12 weeks with ointment containing *Psoralea corylifolia* seed powder (10% *w*/*w*). Ointment could be an effective monotherapy for small circular white lesions of vitiligo [135].

Three studies involved oral administration of plant formulations in forms of syrup, tablets or infusion. Aafi et al. show that extracts of *Ziziphus jujuba* and other herbal plants rich in phenolics and flavonoids, used for 8 weeks by 46 healthy volunteers have a positive effect on patients with facial skin hyperpigmentation and can be used to treat this skin disorder. One daily dose of 7.5 mL of syrup containing *Ziziphus jujuba* was applied: 30% *wt*/*wt*, *Berberis vulgaris*: 10% *wt*/*wt*, *Rhus coriaria* 10% *wt*/*wt*, *Prunus domestica* 7% *wt*/*wt*, and *Rosa damascene* 3% *wt*/*wt* [136]. In addition, Cloucci et al. indicate that combination of *Phyllanthus emblica* extract and vitamin E and caroteinods showed a valuable instrument to increase the effectiveness of vitiligo treatments. Sixty-five subjects treated with one tablet of an oral supplement containing *Phyllanthus emblica* (100 mg), vitamin E (10 mg), and carotenoids (4.7 mg) three times/day for 6 months had a mild repigmentation on the head/neck regions and on the trunk [137]. Resende et al. revealed that combination of *Solanum paniculatum*, *Jacaranda brasiliensis* and *Sonchus oleraceus* extracts showed beneficial properties in the therapy of patients with vitiligo. Consumption of a 800 mL infusion per day (concentration of 15 g of each plant, in total 45 g/2 L) for one year resulted in depigmentation, manifesting with 80% or more of depigmented patches [138].

## 8. In Vitro Studies

Detailed analyses of the modulation of melanogenesis upon treatment with plant extracts and isolated plant compounds in in vitro model were presented in our previous study [60]. However, selected investigations of plant extracts and pure plant compounds on in vivo models, given above, were also conducted on melanocyte monocultures. They included an analysis of gene expression and signaling pathways modulation after treatment.

It was revealed that *Cyrtomium fortunei* [109], *Panax ginseng* [116], *Artocarpus chama* [72] and *Punica granatum* [124] extracts suppress cellular melanogenesis. *Lespedeza bicolor* [112] and *Rhododendron schlippenbachii* [113] extracts show the opposite effect. *Rosa gallica* and *Aster yomena* extracts evoke skin whitening by modulation of MAPKs [126] and ERK and AKT [130] signaling. An opposite response via modulation of p38 signaling was observed after application of *Baccharoides anthelmintica* [118] and *Nelumbo nucifera* [110] extracts. The first one exerts an anti-melanogenic effect, whereas the other one-melanogenic effect. Regulation of cAMP/CREB/MAPKs/MITF and PKA/CREB/MITF axis and suppression of melanogenesis takes place by *Nymphaea nouchali* [107] and *Nelumbo nucifera* [106] extracts. *Pueraria montana var. lobata* exhibits a similar effect by modulation the AKT/GSK3β axis [104].

It was revealed that demethyleugenol β-D-glucopyranoside [85] and epicatechin-3-gallate [100] modulate the expression of MITF, TYR, TYRP-1 or TYRP-2. In addition, linolenic acid and linoleic acid significantly inhibit melanin production by blocking MRC1 signaling [77]. Arctigenin reduce the cellular cAMP level followed by supression of melanin production [86]. The cAMP-CERB-MITF axis is inhibited by jujuboside B, epiceanothic acid and 6‴-feruloylspinosin [102] and melanogenesis is limited. Sweroside [94] and isomaltol glycoside [97] influenced AKT and ERK activation and decrease melanin synthesis.

The detailed information about the impact of given above plant extracts and pure compounds on melanocytes biology are presented in Table 3 and Table 4. According to that studies, analyzed extracts and pure compounds were noncytotoxic towards melanocytes in the range of tested concentration.

## 9. The Patented Plant-Based Compositions and Their Role in the Therapy of Hyperpigmentation and Hypopigmentation

Inventions in the field of cosmetics, including plant-based compositions of topically applied skin care products, effective in reducing and enhancing pigmentation are presented in Table 5 and Table 6.

Some patented formulations contain also pure compounds isolated from plants. For example, the effect of skin lightening was exhibited for rebaudioside A, a diterpene glycoside obtained from Stevia genus (WO2014132217A1), B salvianolic acid, a phenolic acid from *Salviae miltiorrhizae*, (CN102895308A) and madecassoside, a triterpenoid saponin from *Centella asiatica* (WO2014003224A1). In contrast, silybin, a flavonoid from *Silybum marianum* shows stimulating properties for skin pigmentation (US8569358B2).

In addition, an invention entitled “Methods useful in studying or modulating skin or hair pigmentation, plant extracts used in compositions and cosmetic care method” (US8409633B2) presents methods useful in modulation of skin pigmentation. According to its inventors, myosin-X (Myo X) is a crucial protein that controls melanin transfer from melanocytes to keratinocytes. Therefore, compounds that have the ability to impact the expression and activation of Myo X can modulate the skin tone. Accordingly, the molecules that decrease expression of Myo X may reduce skin pigmentation, whereas molecules that increase expression of Myo X may enhance skin pigmentation. The first groups include extracts of the following genus plants: Artocarpus, Cyathea, Secale, Thalassiosira and Buddleja. The second group includes: a soybean extract, preferably a soy seed extract, more preferably a soy seed pericarp extract.

## 10. Conclusions

This literature review presents the impact of plant extracts or pure compounds on melanogenesis potential in in vivo models. Most of the reviewed studies focus on the whitening potential of plants. A relatively large number of studies were performed on the zebrafish model. This organism was used to evaluate the melanogenic potential of both plant extracts and pure compounds. Fewer studies were performed on rodent and human models and were mainly focused on the analysis of plant extracts. In rodent models, plant extracts were administered orally or topically to the skin, and with regard to the human model, they were administered mainly topically. In addition, the majority of patented plant-based compositions was formulations containing plant extracts, applied topically to the skin. The authors evaluated extracts isolated from plants belonging to different species and families as well as pure compounds belonging to different chemical classes. *Panax ginseng* plant, which exhibits significant whitening activity, was the most often studied in various model systems. In contrast, *Salvia miltiorrhiza* extract repeatedly added to patented plant-based formulations, demonstrated hypopigmentation properties. Selected investigations of plant extracts and pure plant compounds on the vivo models were also conducted on melanocyte monocultures. It was reveald that many of phytochemicals are able to modulate different cellular signaling pathways, including those which regulate the expression and activity of melanogenesis-related proteins. In conclusion, molecules of natural origin may play an important role in the therapy of dermatological disorders, including hyperpigmentation and hypopigmentation.

## Figures and Tables

**Table 1:** Melanogenic potential of plant extracts evaluated on the zebrafish model.

Name of the Species/ Family	Part of the Plant	Type of Solvent	Compounds Identified in Extract	Concentration	Incubation Time	Effect	Ref.
*Acalypha indica* L./Euphorbiaceae	whole plant	methanol–ethyl acetate fraction	dioctyl phthalate, (-)-erythromycin, rhamnetin, berberine, keracyanin, spectinomycin, andrographolide, methyl caffeate	10–100 µg/mL	48 hpf	antimelanogenic activity	[71]
*Artocarpus chama* Buch.-Ham./Moraceae	stem	methanol, water	-	200 μg/mL	9–72 hpf	antimelanogenic activity	[72]
*Bletilla striata* (Thunb.) Rchb.f./Orchidaceae	roots, tuber	ethanol	39 chemical compositions, including 24 stilbenoids	10 and 30 mg/mL	6–72 hpf	antimelanogenic activity	[73]
*Blumea balsamifera* (L.) DC./Asteraceae	leaves	ethanol–ethyl acetate fraction	-	10–300 μg/mL	9–57 hpf	antimelanogenic activity	[74]
*Dioscorea nipponica* Makino/Dioscoreaceae	bark	ethanol–methanol fraction	-	6.25–25 μg/mL	9–72 hpf	antimelanogenic activity	[75]
*Elaeocarpus serratus* L./Elaeocarpaceae	leaf	ethanol	gallic acid, myricetin, mearnsetin	50 µg/mL	9–57 hpf	antimelanogenic activity	[76]
*Hosta longipes* (Franch. and Sav.) Matsum./Asparagaceae	whole plant	ethanol–hexane fraction	linolenic acid and linoleic acid	1 μg/mL	9–81 hpf	antimelanogenic activity	[77]
*Morus alba* L./Moraceae	wood	methanol	oxyresveratrol, kuwanon C, mulberroside A, resorcinol, dihydrooxyresveratol, trans-dihydromorin, 2,4,3′-trihydroxydihydrostilbene, kuwanon H, 2,4-dihydroxybenzaldehyde, morusin, moracin M and kuwanon G	70 μg/mL	24–48 hpf	antimelanogenic activity	[78]
*Panax ginseng* C.A.Mey./Araliaceae	whole plant	methanol–water fraction	-	12.5–50 μg/mL	7–72 hpf	antimelanogenic activity	[79]
*Pistacia vera* L./Anacardiaceae	hulls	methanol:water:acetic acid	cyanidin-3-*O*-galactoside, cyanidin-3-*O*-glucoside	2.5–10 μg/mL	24–72 hpf	antimelanogenic activity	[80]
*Reynoutria multiflora* (Thunb.) Moldenke/Polygonaceae	root	water	-	87.5 mg/L	3–4 hpf–4 dpf	antimelanogenic activity	[81]
*Rhanterium suaveolens* Desf./Asteraceae	flowers	methanol	flavonoids and hydroxycinnamic acids	0.5 and 1 mg/mL	24–47 hpf	antimelanogenic activity	[82]
*Senna alata* (L.) Roxb./Fabaceae	leaf	methanol	-	50 μg/mL and 100 μg/mL	9–55 hpf	antimelanogenic activity	[83]
*Sonneratia alba* Sm./Lythraceae	bark	methanol	-	50 µg/mL and 100 µg/ml	9–55 hpf	antimelanogenic activity	[84]
*Streblus taxoides* (B.Heyne ex Roth) Kurz/Moraceae	wood	ethyl acetate, methanol	-	50 μg/mL	9–72 hpf	antimelanogenic activity	[72]

**Table 2:** Melanogenic potential of plant pure compounds evaluated on the zebrafish model.

Name of the Species/Family	Part of the Plant	Identified Compounds	Chemical Class of the Compounds	Concentration	Incubation Time	Effect	Ref.
*Agastache rugosa* (Fisch. and C.A.Mey.) Kuntze/Lamiaceae	leaves	demethyleugenol β-D-glucopyranoside	glucopyranoside	5–30 μM	9–72 hpf	antimelanogenic activity	[85]
*Arctium lappa* L./Asteraceae	seed	arctigenin	lignan	10 μM	15–40 hpf	antimelanogenic activity	[86]
*Artemisia capillaris* Thunb./Asteraceae	leaves and stems	4,5-*O*-dicaffeoylquinic acid	polyphenol	25 μg/mL	9–72 hpf	antimelanogenic activity	[87]
*Artemisia capillaris* Thunb./Asteraceae	-	isofraxidin 7-*O*-(6′-*O*-p-coumaroyl)-β-glucopyranoside	glucopyranoside	12.5 and 25 μg/mL	9–72 hpf	melanogenic enhancer	[88]
*Conioselinum anthriscoides* (H.Boissieu) Pimenov and Kljuykov/Apiaceae	rhizoma	neocnidilide	gamma-lactone	10–20 μM	7–72 hpf	antimelanogenic activity	[89]
*Elaeocarpus serratus* L./Elaeocarpaceae	leaves	gallic acid, myricetin, mearnsetin	phenolic acid, flavone, *O*-methylated flavonol	50 μM	9–57 hpf	antimelanogenic activity	[76]
*Eurya emarginata* (Thunb.) Makino/Pentaphylacaceae	root	rengyolone	cyclohexylethanoid	16.2 and 32.5 μM	9–48 hpf	antimelanogenic activity	[90]
*Hosta longipes* (Franch. and Sav.) Matsum./Asparagaceae	whole plant	linolenic acid and linoleic acid	fatty acids	1 μg/mL	9–81 hpf	antimelanogenic activity	[77]
*Inula japonica* Thunb./Asteraceae	flowers	inularin	sesquiterpene	10–100 μM	10–48 hpf	antimelanogenic activity	[91]
*Inula japonica* Thunb./Asteraceae	flowers	6-*O*-Isobutyrylbritannilactone	lactone	10–100 μM	10–48 hpf	antimelanogenic activity	[92]
*Juniperus communis* L./Cupressaceae	fruits	hypolaetin-7-*O*-β-D-xyloside	flavonoid	1–400 μg/mL	9–72 hpf	antimelanogenic activity	[93]
*Lonicera japonica* Thunb./Caprifoliaceae	whole plant	sweroside	iridoid glycoside	150 and 300 μM	9–72 hpf	antimelanogenic activity	[94]
*Morus alba* L./Moraceae	wood	oxyresveratrol	stilbenoid	50 μg/mL	24–48 hpf	antimelanogenic activity	[78]
*Panax ginseng* C.A.Mey./Araliaceae	berry	floralginsenoside A	ginsenoside	40–160 μg/mL	9–72 hpf	antimelanogenic activity	[95]
*Panax ginseng* C.A.Mey./Araliaceae	leaves	picrionoside A	glucoside	40 and 80 μg/mL	9–72 hpf	antimelanogenic activity	[96]
*Panax ginseng* C.A.Mey./Araliaceae	roots	isomaltol glycoside	glycoside	50 and 100 μg/mL	9–72 hpf	antimelanogenic activity	[97]
*Panax ginseng* C.A.Mey./Araliaceae	leaves	Rh23	ginsenoside	40 and 80 μM	9–72 hpf	antimelanogenic activity	[98]
*Panax ginseng* C.A.Mey./Araliaceae	aerial parts	Rh6, R4, R13	ginsenosides	80 μM	9–72 hpf	antimelanogenic activity	[99]
*Persicaria amphibia* (L.) Delarbre/Polygonaceae		epicatechin-3-gallate	catechin	50–200 μM	24–48 hpf	antimelanogenic activity	[100]
*Viscum album* L./Santalaceae	whole plant	velutin	flavonoid	30 and 300 μg/mL	5–30 hpf	antimelanogenic activity	[101]
*Ziziphus jujuba* Mill./Rhamnaceae	seeds	jujuboside B, epiceanothic acid, 6‴-feruloylspinosin	flavonoid glycosides	20 μM	48–72 hpf	antimelanogenic activity	[102]

**Table 3:** The effects of selected plant extracts on melanocytes biology.

Name of the Species	Type of Cell	Tested Concentration	Biological Properties	Ref.
*Artocarpus* chama Buch.-Ham./Moraceae	B16 melanoma cells	100 μg/mL	TYR and mela-nin biosynthe-sis inhibitory effect	[72]
*Aster yomena* (Kitam.) Honda/Asteraceae	B16 melanoma cells	15–120 µg/mL	Melanin biosynthesis inhibitory effect, modulation of CREB, MITF, TYRP1, and TYRP2 expression	[130]
*Cyrtomium fortunei* J.Sm./Polypodiaceae	Melan-a cells	100 μg/mL	TYR and melanin biosynthesis inhibitory effect	[109]
*Lespedeza bicolor* Turcz./Fabaceae	B16 melanoma cells	5–20 µg/mL	TYR and melanin biosynthesis activatory effect	[112]
*Nelumbo nucifera* Gaertn./Nelumbonaceae	B16 melanoma cells	10–20 µg/mL	TYR and melanin biosynthesis inhibitory effect, modulation of TYRP1 expression	[110]
*Nelumbo nucifera* Gaertn./Nelumbonaceae	B16 melanoma cells	0.3–0.5 mg/mL	TYR and mela-nin biosynthe-sis inhibitory effect, modulation of TYRP1 and MITF expression	[106]
*Nymphaea nouchali* Burm.f./Nymphaeaceae	Melan-a cells	3–30 µg/mL	TYR and melanin biosynthesis inhibitory effect, modulation of TYRP1, TYRP-2 and MITF expression	[107]
*Panax ginseng* C.A.Mey./Araliaceae	B16 melanoma cells	500 μg/mL and 1 mg/mL	TYR and melanin biosynthesis inhibitory effect	[116]
*Pueraria montana var. lobata* (Willd.) Maesen and S.M.Almeida ex Sanjappa and Predeep/Fabaceae	B16 melanoma cells	10–100 µg/mL	modulation of TYR and TYRP1 expression	[104]
*Punica granatum* L./Lythraceae	B16 melanoma cells	0.02 mg/mL	TYR and melanin biosynthesis inhibitory effect	[124]
*Rhododendron schlippenbachii* Maxim./Ericaceae	B16 melanoma cells	5–20 µg/mL	TYR and melanin biosynthesis activatory effect	[113]
*Rosa gallica* L./Rosaceae	B16 melanoma cells	50–200 µg/mL	TYR and melanin biosynthesis inhibitory effect, modulation of ERK, JNK, p38 and MITF expression	[126]
*Baccharoides anthelmintica* (L.) Moench/Asteraceae	B16 melanoma cells	1–5 µg/mL	TYR and melanin biosynthesis activatory effect, modulation of MITF expression	[118]

**Table 4:** The effects of selected plant pure compounds on melanocytes biology.

Name of the Pure Compounds	Type of Cell	Tested Concentration	Biological Properties	Ref.
arctigenin	B16 melanoma cells		TYR and mela-nin biosynthe-sis inhibitory effect	[86]
demethyleugenol β-D-glucopyranoside	Melan-a cells	5–10 µg/mL	TYR and melanin biosynthesis inhibitory effect, modulation of TYRP1 and MITF expression	[85]
epicatechin-3-gallate	B16 melanoma cells	25–200 µM	TYR and melanin biosynthesis inhibitory effect, modulation of TYRP1, TYRP2 and MITF expression	[100]
isomaltol glycoside	B16 melanoma cela	25–100 µg/mL	TYR and mela-nin biosynthe-sis inhibitory effect, modulation of TYRP1 and TYRP2 and MITF expres-sion	[97]
jujuboside B, epiceanothic acid and 6‴-feruloylspinosin	B16 melanoma cells	20 µM	TYR and melanin biosynthesis inhibitory effect, modulation of MITF expression	[102]
linolenic acid and linoleic acid	B16 melanoma cells	10–100 nM	TYR and mela-nin biosynthe-sis inhibitory effect	[77]
sweroside	Melan-a cells	300 μM	TYR and mela-nin biosynthe-sis inhibitory effect, modulation of TYRP1 and TYRP2 expres-sion	[94]

**Table 5:** Patented topical anti-hyperpigmentation plant-based compositions.

Name of the Species/Part of the Plants	Number of Patents
*Alpinia officinarum*–rhizome, *Physalis angulate*–leaves, stems and roots, *Bidens pilosa*–leaves, stems and roots, *Achyrocline satureioides*–flowers	US20170100326A1
*Amorphophallus konjac*–tuber	US20160184218A1
Aspalathus Linearis, Matricaria, Saxifraga, Astragalus, Taraxacum officinale/mongolicum, Ferula varia/foetida, Carthamus tinctorius, Sophora flavescentis, or Chrysanthemum morifolium	US9801809B2
Atractylodis macrocephalae rhizoma, Glycyrrhizae radix et rhizoma, Angelicae sinensis radix, Paconiae radix alba and Poria	US9511013B2
banyan tree, lotus, and clover serum fractions	WO2012115949A3
*Bellis perennis*–flowers	EP1737538B1
*Bellis perennis*–whole plant or flower heads	EP1737538B1
*Butea monosperma* (Identified compound: butrin)	US9775797B2
*Caesalpinia spinosa*–root, stem, leaf, flower, fruit and fruit pod	US20130302265A1
*Caragana sinica*–roots	WO2017003190A1
*Coffea arabica*–roasted beans	CN103517699A
Cortex Mori, Radix Angelicae Dahuricae, Cortex Lycii, Aloe, Flos Rosae Rugosae, Semen Coicis, Herba Artemisiae Scopariae, Folium Eriobotryae, Mel	CN103655388A
*Dipterocarpus intricatus*–leaves	WO2017086692A1
*Fabiana imbricata*–whole plant, or its portion, including stems, leaves, nectar and/or flower petals	US20150342853A1
Ficus serum fraction derived from fresh Ficus cell juice of fresh Ficus leaves	US20120201768A1
*Glycyrrhiza glabra*, *Rubia cordifolia*, *Symplocos racemosa*, *Terminalia arjuna*, *Myristica fragrans*–barks, roots, tubers, stigma, kernels, exudates, stolons, rhizome, leaves, seeds, nuts, berries, fruits, stems and flowers	WO2017072668A1
*Greyia radlkoferi*–leaves (Identified compounds: 5,7-dihidroxyflavone[(2S)-pinocembrin]; 2′, 6′-dihydroxy-4′-methoxydihydrochalcone; 2′,4′,6′-trihydroxyhydrochalcone; 3,5,7-trihydroxyflavone and 4′,5′7-thhydroxyisoflavone)	US20150118337A1
*Lilium candidum*–bulb	US8481093B2
Morus plant leaves	US8980343B2
*Phaseolus vulgaris*–dried navy (haricot)-beans	US9138401B2
*Phaseolus vulgaris*–navy bean	US8747926B2
*Phyllanthus embilica*–fruits, *Bellis perrenis*–flowers, *Glycyrrhiza glabra*–roots	US20160074316A1
*Piper longum*–roots	WO2014014515A2
*Quassia undulata*–leaves	US20160074314A1
*Rhododendron moulmainense*–whole plant	US9333167B2
*Salvia hispanica*–seeds	US8916212B2

**Table 6:** Patented topical anti-hypopigmentation plant-based compositions.

Name of the Species/Part of the Plants	Number of Patents
*Coreopsis tinctoria*	CN104523796A
Cortex Dictamni, Rhizoma Gastrodiae, Flos Carthami, Fructus Psoraleae, Radix Salviae Miltiorrhizae, Radix Angelicae Dahuricae, Flos Lonicerae, Fructus Mume, Radix Astragali, Borneolum Syntheticum, Scorpio, Fructus Lycii, Radix Saposhnikoviae	CN104474298A
Folium Fici, Herba Spirodelae and Pericarpium Citri junoris	CN102274359B
Folium Ginkgo, Herba Spirodelae, Pericarpium Citri junoris	CN102846500A
Fructus Mume, Fructus Psoraleae, Rhizoma Zingiberis Recens,	CN104338104A
Fructus Mume, Fructus Psoraleae, Rhizoma Zingiberis Recens,	CN104338104A
Fructus Tribuli, Herba speranskiae tuberculatae, hair Rhizoma Zingiberis Recens, Cortex Cinnamomi, Fructus Psoraleae, Radix Polygoni Multiflori Preparata, Radix Salviae Miltiorrhizae, Radix Arnebiae (Radix Lithospermi)	CN105535128A
*Gynostemma pentaphyllum* (Isolated compounds: saponins)	US20150209376A1
Herba Schizonepetae, Radix Saposhnikoviae, Herba Menthae, Scorpio, Periostracum Cicadae, Radix Ginseng, Radix Astragali, Semen Persicae, Radix Angelicae Sinensis, Rhizoma Chuanxiong, Radix Paeoniae Alba, Radix Rehmanniae Preparata, Radix Notoginseng, Fructus Ligustri Lucidi, Herba Ecliptae, Fructus Psoraleae, Radix Angelicae Dahuricae, Stigma Croci, Fructus Fici, Radix Arnebiae (Radix Lithospermi), Semen Astragali Complanati, Fructus Cnidii, Radix Polygoni Multiflori, Fructus Mume, Borneolum Syntheticum, Flos Caryophylli	CN103385986A
Herba Taraxaci, Radix Rehmanniae, Flos Primulae Vittatae, Radix Salviae Miltiorrhizae, Stigma Croci	CN104274628A
*Pueraria genus*–roots (Identified ingredient: puerarin)	US20120329739A1
Radix Rehmanniae, Radix Angelicae Sinensis, Ramulus Cinnamomi, Rhizoma Chuanxiong, Flos Carthami, Cortex Moutan, Cortex Dictamni, Radix Polygoni Multiflori, Fructus Tribuli, Herba Ephedrae, Radix Euphorbiae Fischerianae (Radix Euphorbiae Ebracteolatae), Radix Cyathulae, Fructus Cnidii, Periostracum Cicadae, Flos Lonicerae, Spina Gleditsiae, Radix Saposhnikoviae, Radix Sophorae Flavescentis, Rhizoma Atractylodis, Semen Astragali Complanati, Fructus Ligustri Lucidi, sub-lotus grass, Radix Notoginseng	CN104257857A
*Sophora japonica*	US8673371B2

## Data Availability

Not applicable.

## References

[1] Boer M., Duchnik E., Maleszka R., Marchlewicz M. (2016). Structural and biophysical characteristics of human skin in maintaining proper epidermal barrier function. Postep. Dermatol. Alergol..

[2] Cichorek M., Wachulska M., Stasiewicz A., Tymińska A. (2013). Skin melanocytes: Biology and development. Postep. Dermatol. Alergol..

[3] Maranduca M.A., Branisteanu D., Serban D.N., Branisteanu D.C., Stoleriu G., Manolache N., Serban I.L. (2019). Synthesis and physiological implications of melanic pigments (review). Oncol. Lett..

[4] Nicolaidou E., Katsambas A.D. (2014). Pigmentation disorders: Hyperpigmentation and hypopigmentation. Clin. Dermatol..

[5] Dawid-Pać R. (2013). Medicinal plants used in treatment of inflammatory skin diseases. Postep. Dermatol. Alergol..

[6] Tabassum N., Hamdani M. (2014). Plants used to treat skin diseases. Pharmacogn. Rev..

[7] Hussein A.R., El-Anssary A., Builders P.F. (2019). Plants Secondary Metabolites: The Key Drivers of the Pharmacological Actions of Medicinal Plants. Herbal Medicine.

[8] Videira I.F.D.S., Moura D.F.L., Magina S. (2013). Mechanisms regulating melanogenesis. An. Bras. Dermatol..

[9] D’Mello S.A.N., Finlay G.J., Baguley B.C., Askarian-Amiri M.E. (2016). Signaling pathways in melanogenesis. Int. J. Mol. Sci..

[10] Ali S.A., Naaz I., Sakuma K. (2015). Current Challenges in Understanding the Story of Skin Pigmentation—Bridging the Morpho-Anatomical and Functional Aspects of Mammalian Melanocytes. Muscle Cell and Tissue.

[11] Tsatmali M., Ancans J., Thody A.J. (2002). Melanocyte function and its control by melanocortin peptides. J. Histochem. Cytochem..

[12] Wang J.X., Fukunaga-Kalabis M., Herlyn M. (2016). Crosstalk in skin: Melanocytes, keratinocytes, stem cells, and melanoma. J. Cell Commun. Signal..

[13] Wang Y., Viennet C., Robin S., Berthon J.Y., He L., Humbert P. (2017). Precise role of dermal fibroblasts on melanocyte pigmentation. J. Dermatol. Sci..

[14] Solano F. (2014). Melanins: Skin Pigments and Much More—Types, Structural Models, Biological Functions, and Formation Routes. New J. Sci..

[15] Hearing V.J. (2011). Milestones in Melanocytes/Melanogenesis. J. Invest. Dermatol..

[16] Silver D.L., Hou L., Pavan W.J. (2006). The genetic regulation of pigment cell development. Adv. Exp. Med. Biol..

[17] Kus N.J., Dolinska M.B., Young K.L., Dimitriadis E.K., Wingfield P.T., Sergeev Y.V. (2018). Membrane-associated human tyrosinase is an enzymatically active monomeric glycoprotein. PLoS ONE.

[18] Lai X., Wichers H.J., Soler-Lopez M., Dijkstra B.W. (2018). Structure and Function of Human Tyrosinase and Tyrosinase-Related Proteins. Chem. Eur. J..

[19] Otręba M., Rok J., Buszman E., Wrześniok D. (2012). Regulation of melanogenesis: The role of cAMP and MITF. Postep. Hig. Med. Dosw..

[20] Solano F. (2020). Photoprotection and skin pigmentation: Melanin-related molecules and some other new agents obtained from natural sources. Molecules.

[21] Brenner M., Hearing V.J. (2008). The protective role of melanin against UV damage in human skin. Photochem. Photobiol..

[22] ElObeid A.S., Kamal-Eldin A., Abdelhalim M.A.K., Haseeb A.M. (2017). Pharmacological Properties of Melanin and its Function in Health. Basic Clin. Pharmacol. Toxicol..

[23] Fernie A.R., Trethewey R.N., Krotzky A.J., Willmitzer L. (2004). Metabolite profiling: From diagnostics to systems biology. Nat. Rev. Mol. Cell Biol..

[24] Erb M., Kliebenstein D.J. (2020). Plant Secondary Metabolites as Defenses, Regulators, and Primary Metabolites: The Blurred Functional Trichotomy1[OPEN]. Plant Physiol..

[25] Yeshi K., Crayn D., Ritmejerytė E., Wangchuk P. (2022). Plant Secondary Metabolites Produced in Response to Abiotic Stresses Has Potential Application in Pharmaceutical Product Development. Molecules.

[26] Atanasov A.G., Waltenberger B., Pferschy-Wenzig E.M., Linder T., Wawrosch C., Uhrin P., Temml V., Wang L., Schwaiger S., Heiss E.H. (2015). Discovery and resupply of pharmacologically active plant-derived natural products: A review. Biotechnol. Adv..

[27] Sofowora A., Ogunbodede E., Onayade A. (2013). The role and place of medicinal plants in the strategies for disease prevention. Afr. J. Tradit. Complement. Altern. Med..

[28] Pang Z., Chen J., Wang T., Gao C., Li Z., Guo L., Xu J., Cheng Y. (2021). Linking Plant Secondary Metabolites and Plant Microbiomes: A Review. Front. Plant Sci..

[29] Wink M. (2015). Modes of Action of Herbal Medicines and Plant Secondary Metabolites. Medicines.

[30] Arruda R.L., de Sousa Garcia N.O., Souza N.F., da Silva F.M., Arruda E.L., da Conceição E.C. (2021). Natural photoprotectors: A literature review. Res. Soc. Dev..

[31] Kowalczyk T., Merecz-Sadowska A., Rijo P., Mori M., Hatziantoniou S., Górski K., Szemraj J., Piekarski J., Śliwiński T., Bijak M. (2022). Hidden in Plants—A Review of the Anticancer Potential of the Solanaceae Family in In Vitro and In Vivo Studies. Cancers.

[32] Merecz-Sadowska A., Sitarek P., Kucharska E., Kowalczyk T., Zajdel K., Cegliński T., Zajdel R. (2021). Antioxidant properties of plant-derived phenolic compounds and their effect on skin fibroblast cells. Antioxidants.

[33] Merecz-Sadowska A., Sitarek P., Zajdel K., Kucharska E., Kowalczyk T., Zajdel R. (2021). The modulatory influence of plant-derived compounds on human keratinocyte function. Int. J. Mol. Sci..

[34] Merecz-Sadowska A., Sitarek P., Śliwiński T., Zajdel R. (2020). Anti-inflammatory activity of extracts and pure compounds derived from plants via modulation of signaling pathways, especially PI3K/AKT in macrophages. Int. J. Mol. Sci..

[35] Sitarek P., Kowalczyk T., Wieczfinska J., Merecz-Sadowska A., Górski K., Śliwiński T., Skała E. (2020). Plant extracts as a natural source of bioactive compounds and potential remedy for the treatment of certain skin diseases. Curr. Pharm. Des..

[36] Sitarek P., Merecz-Sadowska A., Kowalczyk T., Wieczfinska J., Zajdel R., Śliwiński T. (2020). Potential synergistic action of bioactive compounds from plant extracts against skin infecting microorganisms. Int. J. Mol. Sci..

[37] Sitarek P., Merecz-Sadowska A., Śliwiński T., Zajdel R., Kowalczyk T. (2020). An in vitro evaluation of the molecular mechanisms of action of medical plants from the lamiaceae family as effective sources of active compounds against human cancer cell lines. Cancers.

[38] Zielinska-Blizniewska H., Sitarek P., Merecz-Sadowska A., Malinowska K., Zajdel K., Jablonska M., Sliwinski T., Zajdel R. (2019). Plant extracts and reactive oxygen species as two counteracting agents with anti- and pro-obesity properties. Int. J. Mol. Sci..

[39] Choquenet B., Couteau C., Paparis E., Coiffard L.J.M. (2008). Quercetin and rutin as potential sunscreen agents: Determination of efficacy by an in vitro method. J. Nat. Prod..

[40] Kazumy De Lima Yamaguchi K., Dos L., Santarém S., Lamarão C.V., Lima E.S., Florêncio Da Veiga-Junior V. (2016). Avaliação in vitro da Atividade Fotoprotetora de Resíduos de Frutas Amazônicas. Sci. Amaz..

[41] Velasco M.V.R., Balogh T.S., Pedriali C.A., Sarruf F.D., Pinto C.A.S.O., Kaneko T.M., Baby A.R. (2008). Rutin association with ethylhexyl methoxycinnamate and benzophenone-3: In vitro evaluation of the photoprotection effectiveness by reflectance spectrophotometry. Lat. Am. J. Pharm..

[42] Choquenet B., Couteau C., Paparis E., Coiffard L.J.M. (2009). Flavonoids and polyphenols, molecular families with sunscreen potential: Determining effectiveness with an in vitro method. Nat. Prod. Commun..

[43] Stevanato R., Bertelle M., Fabris S. (2014). Photoprotective characteristics of natural antioxidant polyphenols. Regul. Toxicol. Pharmacol..

[44] Gangwar V., Garg A., Lomore K., Korla K., Bhat S.S., Rao R.P., Rafiq M., Kumawath R., Uddagiri B.V., Kareenhalli V.V. (2021). Immunomodulatory effects of a concoction of natural bioactive compounds—Mechanistic insights. Biomedicines.

[45] Jalali A., Zarshenas M.M. (2021). AKT/GSK-3 Pathway Targeting; Botanicals and Bioactive Compounds with Anticancer Activities. Curr. Pharm. Des..

[46] Bang J., Zippin J.H. (2021). Cyclic adenosine monophosphate (cAMP) signaling in melanocyte pigmentation and melanomagenesis. Pigment Cell Melanoma Res..

[47] Mosca S., Cardinali G., Flori E., Briganti S., Bottillo I., Mileo A.M., Maresca V. (2021). The PI3K pathway induced by αMSH exerts a negative feedback on melanogenesis and contributes to the release of pigment. Pigment Cell Melanoma Res..

[48] Wang C., Zhao L., Su Q., Fan X., Wang Y., Gao S., Wang H., Chen H., Chan C.B., Liu Z. (2016). Phosphorylation of MITF by AKT affects its downstream targets and causes TP53-dependent cell senescence. Int. J. Biochem. Cell Biol..

[49] Naqvi S., Martin K.J., Arthur J.S.C. (2014). CREB phosphorylation at Ser133 regulates transcription via distinct mechanisms downstream of cAMP and MAPK signalling. Biochem. J..

[50] Zhang J., Bui T.N., Xiang J., Lin A. (2006). Cyclic AMP Inhibits p38 Activation via CREB-Induced Dynein Light Chain. Mol. Cell. Biol..

[51] Li P.H., Chiu Y.P., Shih C.C., Wen Z.H., Ibeto L.K., Huang S.H., Chiu C.C., Ma D.L., Leung C.H., Chang Y.N. (2016). Biofunctional Activities of Equisetum ramosissimum Extract: Protective Effects against Oxidation, Melanoma, and Melanogenesis. Oxid. Med. Cell. Longev..

[52] Terazawa S., Imokawa G. (2018). Signaling Cascades Activated by UVB in Human Melanocytes Lead to the Increased Expression of Melanocyte Receptors, Endothelin B Receptor and c-KIT. Photochem. Photobiol..

[53] Teng Y., Fan Y., Ma J., Lu W., Liu N., Chen Y., Pan W., Tao X. (2021). The pi3k/akt pathway: Emerging roles in skin homeostasis and a group of non-malignant skin disorders. Cells.

[54] Inamdar G.S., Madhunapantula S.R.V., Robertson G.P. (2010). Targeting the MAPK pathway in melanoma: Why some approaches succeed and other fail. Biochem. Pharmacol..

[55] Phung B., Sun J., Schepsky A., Steingrimsson E., Rönnstrand L. (2011). C-KIT signaling depends on microphthalmia-associated transcription factor for effects on cell proliferation. PLoS ONE.

[56] Alexeev V., Yoon K. (2006). Distinctive role of the cKit receptor tyrosine kinase signaling in mammalian melanocytes. J. Investig. Dermatol..

[57] Sun Q., Rabbani P., Takeo M., Lee S.H., Lim C.H., Noel E.N.S., Taketo M.M., Myung P., Millar S., Ito M. (2018). Dissecting Wnt Signaling for Melanocyte Regulation during Wound Healing. J. Investig. Dermatol..

[58] Schepsky A., Bruser K., Gunnarsson G.J., Goodall J., Hallsson J.H., Goding C.R., Steingrimsson E., Hecht A. (2006). The Microphthalmia-Associated Transcription Factor Mitf Interacts with β-Catenin To Determine Target Gene Expression. Mol. Cell. Biol..

[59] Swope V.B., Abdel-Malek Z.A. (2016). Significance of the melanocortin 1 and endothelin B receptors in melanocyte homeostasis and prevention of sun-induced genotoxicity. Front. Genet..

[60] Merecz-Sadowska A., Sitarek P., Kowalczyk T., Zajdel K., Kucharska E., Zajdel R. (2022). The Modulation of Melanogenesis in B16 Cells Upon Treatment with Plant Extracts and Isolated Plant Compounds. Molecules.

[61] Plensdorf S., Livieratos M., Dada N. (2017). Pigmentation Disorders: Diagnosis and Management. Am. Fam. Physician.

[62] Rathee P., Kumar S., Kumar D., Kumari B., Yadav S.S. (2021). Skin hyperpigmentation and its treatment with herbs: An alternative method. Future J. Pharm. Sci..

[63] Kanlayavattanakul M., Lourith N. (2018). Plants and Natural Products for the Treatment of Skin Hyperpigmentation—A Review. Planta Med..

[64] Pang Y., Wu S., He Y., Nian Q., Lei J., Yao Y., Guo J., Zeng J. (2021). Plant-Derived Compounds as Promising Therapeutics for Vitiligo. Front. Pharmacol..

[65] Hussain I. (2021). The safety of medicinal plants used in the treatment of vitiligo and hypermelanosis: A systematic review of use and reports of harm. Clin. Cosmet. Investig. Dermatol..

[66] Teame T., Zhang Z., Ran C., Zhang H., Yang Y., Ding Q., Xie M., Gao C., Ye Y., Duan M. (2019). The use of zebrafish (Danio rerio) as biomedical models. Anim. Front..

[67] Cline A., Feldman S.R. (2016). Zebrafish for modeling skin disorders. Dermatol. Online J..

[68] Naomi R., Bahari H., Yazid M.D., Embong H., Othman F. (2021). Zebrafish as a model system to study the mechanism of cutaneous wound healing and drug discovery: Advantages and challenges. Pharmaceuticals.

[69] Russo I., Sartor E., Fagotto L., Colombo A., Tiso N., Alaibac M. (2022). The Zebrafish model in dermatology: An update for clinicians. Discov. Oncol..

[70] Neuffer S.J., Cooper C.D. (2022). Zebrafish Syndromic Albinism Models as Tools for Understanding and Treating Pigment Cell Disease in Humans. Cancers.

[71] Kalaivani D., Arun V. (2022). Anti-melanogenic Potential of Acalypha indica Ethyl Acetate Fraction on Zebra fish Embryos. Curr. Trends Biotechnol. Pharm..

[72] Dej-adisai S., Parndaeng K., Wattanapiromsakul C., Nuankaew W., Kang T. (2019). Effects of selected moraceae plants on tyrosinase enzyme and melanin content. Pharmacogn. Mag..

[73] Luo Y., Wang J., Li S., Wu Y., Wang Z., Chen S., Chen H. (2022). Discovery and identification of potential anti-melanogenic active constituents of Bletilla striata by zebrafish model and molecular docking. BMC Complement. Med. Ther..

[74] Thach B.Đ., Vu Q., Dao T., Thi L., Giang L., Nguyen T., Linh T., Thi B., Pham N., Uyen A. (2017). Inhibitor effect of flavonoid from blumea balsamifera (l.) dc. leaves extract on melanin synthesis in cultured B16F10 cell line and zebrafish. Eur. J. Res. Med. Sci..

[75] Ding H.Y., Lin P.W., Wang H.W., Chang T.S. (2014). Methanol partition fraction of ethanol extract of discorea nipponica makino inhibits melanogenesis. Trop. J. Pharm. Res..

[76] Huang C.Y., Liu I.H., Huang X.Z., Chen H.J., Chang S.T., Chang M.L., Ho Y.T., Chang H.T. (2021). Antimelanogenesis effects of leaf extract and phytochemicals from ceylon olive (*Elaeocarpus serratus*) in zebrafish model. Pharmaceutics.

[77] Lee J.W., Kim Y., Choi S.J., Kim S.H., Ha C.W., Jang S., Chae D., Sung S., Ham J., Sohn E.H. (2021). Hosta longipes inhibits melanogenesis by reducing expression of the melanocortin 1 receptor. Mol. Cell. Toxicol..

[78] Chaita E., Lambrinidis G., Cheimonidi C., Agalou A., Beis D., Trougakos I., Mikros E., Skaltsounis A.L., Aligiannis N., Ferreira I.C.F.R. (2017). Anti-melanogenic properties of Greek plants. A novel depigmenting agent from morus alba wood. Molecules.

[79] Dai Y.L., Yang D., Song L.H., Yang H.M., Yu J.B., Zheng F., Yue H., Chen C.B., Wang E.P. (2021). Low Molecular Weight Oligosaccharide from Panax ginseng C.A. Meyer against UV-Mediated Apoptosis and Inhibits Tyrosinase Activity in Vitro and in Vivo. Evid.-Based Complement. Altern. Med..

[80] Smeriglio A., D’Angelo V., Denaro M., Trombetta D., Germanò M.P. (2021). The Hull of Ripe Pistachio Nuts (*Pistacia vera* L.) as a Source of New Promising Melanogenesis Inhibitors. Plant Foods Hum. Nutr..

[81] Thanh D.T.H., Thanh N.L., Thang N.D. (2016). Toxicological and melanin synthesis effects of Polygonum multiflorum root extracts on zebrafish embryos and human melanocytes. Biomed. Res. Ther..

[82] Chelly S., Chelly M., Occhiuto C., Cimino F., Cristani M., Saija A., Molonia M.S., Ruberto G., D’Angelo V., Germanò M.P. (2021). Evaluation of Antioxidant, Anti-Inflammatory and Antityrosinase Potential of Extracts from Different Aerial Parts of Rhanterium suaveolens from Tunisia. Chem. Biodivers..

[83] Lelina F.M.U., Fuentes R.G. (2020). Cassia alata leaf methanolic extracts decreased melanin pigmentation in Zebrafish. Philipp. J. Nat. Sci..

[84] Grisola M.A.C., Fuentes R.G. (2017). Phenotype-based screening of selected mangrove methanolic crude extracts with anti-melanogenic activity using zebrafish (*Danio rerio*) as a model. ScienceAsia.

[85] Lee T.H., Park S., Yoo G., Jang C., Kim M.H., Kim S.H., Kim S.Y. (2016). Demethyleugenol β-Glucopyranoside Isolated from Agastache rugosa Decreases Melanin Synthesis via Down-regulation of MITF and SOX9. J. Agric. Food Chem..

[86] Park H., Song K.H., Jung P.M., Kim J.E., Ro H., Kim M.Y., Ma J.Y. (2013). Inhibitory effect of arctigenin from fructus arctii extract on melanin synthesis via repression of tyrosinase expression. Evid.-Based Complement. Altern. Med..

[87] Tabassum N., Lee J.H., Yim S.H., Batkhuu G.J., Jung D.W., Williams D.R. (2016). Isolation of 4,5-O-Dicaffeoylquinic Acid as a Pigmentation Inhibitor Occurring in Artemisia capillaris Thunberg and Its Validation In Vivo. Evid.-Based Complement. Altern. Med..

[88] Yim S.H., Tabassum N., Kim W.H., Cho H., Lee J.H., Batkhuu G.J., Kim H.J., Oh W.K., Jung D.W., Williams D.R. (2017). Isolation and Characterization of Isofraxidin 7-O-(6′-O-p-Coumaroyl)-β-glucopyranoside from Artemisia capillaris Thunberg: A Novel, Nontoxic Hyperpigmentation Agent That Is Effective In Vivo. Evid.-Based Complement. Altern. Med..

[89] Cheng M.C., Lee T.H., Chu Y.T., Syu L.L., Hsu S.J., Cheng C.H., Wu J., Lee C.K. (2018). Melanogenesis inhibitors from the rhizoma of ligusticum sinense in B16-f10 melanoma cells in vitro and zebrafish in vivo. Int. J. Mol. Sci..

[90] Kim J.H., Jeong S.C., Hwang J.S., Lee E.S., Lee S.M. (2014). Modulation of melanin synthesis by rengyolone isolated from the root of eurya emarginata in melan-a cells. Phyther. Res..

[91] Jang D.K., Jung S.H., Jeong J.H., Yoo H.M., Lee I.S., Shin H.S. (2020). The Antimelanogenic Effect of Inularin Isolated from Flowers of Inula britannica on B16F10 Melanoma Cells and Zebrafish Embryos. J. Microbiol. Biotechnol..

[92] Jang D.K., Pham C.H., Lee I.S., Jung S.H., Jeong J.H., Shin H.S., Yoo H.M. (2020). Anti-melanogenesis activity of 6-O-isobutyrylbritannilactone from inula britannica on B16F10 melanocytes and in vivo zebrafish models. Molecules.

[93] Jeong E.J., Jegal J., Chung K.W., Noh S.G., Chung H.Y., Nam Y.H., Kang T.H., Kim S.N., Yang M.H. (2017). Hypolaetin-7-O-β-D-xyloside from Juniperus communis fruits inhibits melanogenesis on zebrafish pigmentation. Nat. Prod. Commun..

[94] Jeong Y.T., Jeong S.C., Hwang J.S., Kim J.H. (2015). Modulation effects of sweroside isolated from the Lonicera japonica on melanin synthesis. Chem. Biol. Interact..

[95] Lee D.Y., Lee J., Jeong Y.T., Byun G.H., Kim J.H. (2017). Melanogenesis inhibition activity of floralginsenoside A from Panax ginseng berry. J. Ginseng Res..

[96] Lee D.Y., Jeong S.C., Jeong Y.T., Lee M.K., Seo K.H., Lee J.W., Kim G.S., Lee S.E., Baek N.I., Kim J.H. (2015). Antimelanogenic effects of picrionoside a isolated from the leaves of Korean ginseng. Biol. Pharm. Bull..

[97] Lee S.M. (2019). Antimelanogenic effect of isomaltol glycoside from red ginseng extract. J. Soc. Cosmet. Sci. Korea.

[98] Lee D.Y., Kim H.G., Lee Y.G., Kim J.H., Lee J.W., Choi B.R., Jang I.B., Kim G.S., Baek N.I. (2018). Isolation and quantification of ginsenoside rh23, a new anti-melanogenic compound from the leaves of panax ginseng. Molecules.

[99] Lee D.Y., Cha B.J., Lee Y.S., Kim G.S., Noh H.J., Kim S.Y., Kang H.C., Kim J.H., Baek N.I. (2015). The potential of minor ginsenosides isolated from the leaves of Panax ginseng as inhibitors of melanogenesis. Int. J. Mol. Sci..

[100] Lee Y.K., Hwang B.S., Hwang Y., Lee S.Y., Oh Y.T., Kim C.H., Nam H.J., Jeong Y.T. (2021). Melanogenesis inhibitory activity of epicatechin-3-O-gallate isolated from *Polygonum amphibium* L.. Microbiol. Biotechnol. Lett..

[101] Jung S.H., Kim J., Eum J., Choe J.W., Kim H.H., Kee Y., Lee K. (2019). Velutin, an aglycone extracted from Korean mistletoe, with improved inhibitory activity against melanin biosynthesis. Molecules.

[102] Molagoda I.M.N., Lee K.T., Athapaththu A.M.G.K., Choi Y.H., Hwang J., Sim S.J., Kang S., Kim G.Y. (2021). Flavonoid glycosides from ziziphus jujuba var. Inermis (bunge) rehder seeds inhibit α-melanocyte-stimulating hormone-mediated melanogenesis. Int. J. Mol. Sci..

[103] Dellambra E., Odorisio T., D’Arcangelo D., Failla C.M., Facchiano A. (2019). Non-animal models in dermatological research. ALTEX.

[104] Han E.B., Chang B.Y., Kim D.S., Cho H.K., Kim S.Y. (2014). Melanogenesis inhibitory effect of aerial part of Pueraria thunbergiana in vitro and in vivo. Arch. Dermatol. Res..

[105] Jegal J., Chung K.W., Chung H.Y., Jeong E.J., Yang M.H. (2017). The standardißed extract of juniperus communis alleviates hyperpigmentation in vivo HRM-2 hairless mice and in vitro murine B16 melanoma cells. Biol. Pharm. Bull..

[106] Lai P.-J., Kao E.-S., Chen S.-R., Huang Y.-T., Wang C.-J., Huang H.-P. (2020). *Nelumbo nucifera* Leaf Extracts Inhibit Melanogenesis in B16 Melanoma Cells and Guinea Pigs through Downregulation of CREB/MITF Activation. J. Food Nutr. Res..

[107] Alam M.B., Ahmed A., Motin M.A., Kim S., Lee S.H. (2018). Attenuation of melanogenesis by *Nymphaea nouchali* (Burm. f) flower extract through the regulation of cAMP/CREB/MAPKs/MITF and proteasomal degradation of tyrosinase. Sci. Rep..

[108] Ubaydee A.H.N., Issa R., Hajleh M.N.A., Ghanim B.Y., Al-Akayleh F., Qinna A. (2022). The effect of Medicago sativa extract and light on skin hypopigmentation disorders in C57/BL6 mice. J. Cosmet. Dermatol..

[109] Choi S.Y. (2013). Inhibitory effects of *Cyrtomium fortunei* J. Smith root extract on melanogenesis. Pharmacogn. Mag..

[110] Hsu J.Y., Lin H.H., Li T.S., Tseng C.Y., Wong Y., Chen J.H. (2020). Anti-Melanogenesis Effects of Lotus Seedpod In Vitro and In Vivo. Nutrients..

[111] Moreira C.G., Carrenho L.Z., Pawloski P.L., Soley B.S., Cabrini D.A., Otuki M.F. (2015). Pre-clinical evidences of Pyrostegia venusta in the treatment of vitiligo. J. Ethnopharmacol..

[112] Ha S.Y., Jung J.Y., Kang H.Y., Kim T.-H., Yang J.-K. (2020). Tyrosinase activity and melanogenic effects of Lespedeza bicolor extract in vitro and in vivo. BioResources.

[113] Ha S.Y., Jung J.Y., Kang H.Y., Kim T.H., Yang J.K. (2020). Tyrosinase activity and melanogenic effects of rhododendron schlippenbachii extract in vivo and in vitro. J. Korean Wood Sci. Technol..

[114] Niu Y., Wang S., Li C., Wang J., Liu Z., Kang W. (2020). Effective Compounds From Caesalpinia sappan L. on the Tyrosinase In Vitro and In Vivo. Nat. Prod. Commun..

[115] Hamamoto A., Isogai R., Maeda M., Hayazaki M., Horiyama E., Takashima S., Koketsu M., Takemori H. (2020). The high content of ent-11α-hydroxy-15-oxo-kaur- 16-en-19-oic Acid in *Adenostemma lavenia* (L.) O. kuntze leaf extract: With preliminary in vivo assays. Foods.

[116] Saba E., Kim S.H., Lee Y.Y., Park C.K., Oh J.W., Kim T.H., Kim H.K., Roh S.S., Rhee M.H. (2020). Korean Red Ginseng extract ameliorates melanogenesis in humans and induces antiphotoaging effects in ultraviolet B–irradiated hairless mice. J. Ginseng Res..

[117] Park S.J., Lee M., Yun J.M., Kim D., Lee J., Lee Y.H. (2021). Zingiber mioga extract improves moisturization and depigmentation of skin and reduces wrinkle formation in uvb-irradiated hrm-2 hairless mice. Appl. Sci..

[118] Zang D., Maimaiti Z., Mamat N., Li H., Lu X., Turak A., Luo Y. (2021). Pigmentation Effects of Kaliziri Standard Extracts on Melanocytes, C57BL/6 mice of Hydroquinone-Induced and Guinea Pigs of Hydrogen Peroxide- Induced Hyperpigmentation Model. Res. Sq.

[119] Preedalikit W., Pintha K., Tantipaiboonwong P., Aunsri N., Vivattanaseth P., Mungmai L. (2020). Inhibitory effect of perilla frutescens l. Leaves extract on melanogenesis and skin whitening efficacy in the underarm whitening product application. Key Eng. Mater..

[120] Tilaar A., Ranti A., Mun’im A. (2017). The efficacy study of snake fruit (Salacca edulis Reinw Var. Bongkok) extract as skin lightening agent. Pharmacogn. J..

[121] Whangsomnuek N., Mungmai L., Amornlerdpison D., Mengamphan K. (2019). Efficiency of skin whitening cream containing etlingera elatior flower and leaf extracts in volunteers. Cosmetics.

[122] Omar S.S.S., Hadi H., Mohd Hanif N., Ahmad H.M.A., Ng S.F. (2021). Lightening effect of skin lightening cream containing piper betle l. Extract in human volunteers. Cosmetics.

[123] Mungmai L., Preedalikit W., Pintha K., Tantipaiboonwong P., Aunsri N. (2020). Collagenase and Melanogenesis Inhibitory Effects of Perilla Frutescens Pomace Extract and Its Efficacy in Topical Cosmetic Formulations. Cosmetics.

[124] Kanlayavattanakul M., Chongnativisit W., Chaikul P., Lourith N. (2020). Phenolic-rich Pomegranate Peel Extract: In Vitro, Cellular, and in Vivo Activities for Skin Hyperpigmentation Treatment. Planta Med..

[125] Ayuningtyas I.N., Mun’Im A., Sutriyo S. (2018). The study of safety and skin whitening efficacy of melinjo (*Gnetum gnemon* L.) seed extract-loaded lipid particle gel. Pharmacogn. Res..

[126] Song Y.R., Lim W.C., Han A., Lee M.H., Shin E.J., Lee K.M., Nam T.G., Lim T.G. (2020). Rose Petal Extract (*Rosa gallica*) Exerts Skin Whitening and Anti-Skin Wrinkle Effects. J. Med. Food.

[127] Choi K.T., Kim J.H., Cho H.T., Lim S.S., Kwak S.S., Kim Y.J. (2016). Dermatologic evaluation of cosmetic formulations containing Chrysanthemum indicum extract. J. Cosmet. Dermatol..

[128] Lourith N., Kanlayavattanakul M. (2020). Formulation and clinical evaluation of the standardized Litchi chinensis extract for skin hyperpigmentation and aging treatments. Ann. Pharm. Fr..

[129] Akhtar N., Khan H.M.S., Ashraf S., Mohammad I.S., Ali A. (2014). Skin depigmentation activity of crocus sativus extract cream. Trop. J. Pharm. Res..

[130] Lee J.I., Seo J.H., Ko E.S., Cho S.M., Kang J.R., Jeong J.H., Jeong Y.J., Kim C.Y., Cha J.D., Kim W.S. (2021). Inhibition of melanogenesis by aster yomena callus pellet extract in melanoma cells and patients with skin pigmentation. Int. J. Med. Sci..

[131] Majeed M., Majeed S., Jain R., Mundkur L., Rajalakshmi H.R., Lad P.S., Neupane P. (2020). An open-label single-arm, monocentric study assessing the efficacy and safety of natural pterostilbene (*Pterocarpus marsupium*) for skin brightening and antiaging effects. Clin. Cosmet. Investig. Dermatol..

[132] Ali A., Akhtar N., Khan M.S. (2012). In vivo evaluation: The effects of a cream containing Acacia bark extract on skin melanin and erythema content. Postep. Dermatol. Alergol..

[133] Khan B.A., Akhtar N., Hussain I., Abbas K.A., Rasul A. (2013). Whitening efficacy of plant extracts including Hippophae rhamnoides and Cassia fistula extracts on the skin of Asian patients with melasma. Postep. Dermatol. Alergol..

[134] Mihăilă B., Dinică R.M., Tatu A.L., Buzia O.D. (2019). New insights in vitiligo treatments using bioactive compounds from Piper nigrum. Exp. Ther. Med..

[135] Hussain I., Hussain N., Manan A., Rashid A., Khan B., Bakhsh S. (2016). Fabrication of anti-vitiligo ointment containing Psoralea corylifolia: In vitro and in vivo characterization. Drug Des. Devel. Ther..

[136] Aafi E., Shams Ardakani M.R., Ahmad Nasrollahi S., Mirabzadeh Ardakani M., Samadi A., Hajimahmoodi M., Naeimifar A., Pourjabbar Z., Amiri F., Firooz A. (2022). Brightening effect of Ziziphus jujuba (jujube) fruit extract on facial skin: A randomized, double-blind, clinical study. Dermatol. Ther..

[137] Colucci R., Dragoni F., Conti R., Pisaneschi L., Lazzeri L., Moretti S. (2015). Evaluation of an oral supplement containing Phyllanthus emblica fruit extracts, vitamin E, and carotenoids in vitiligo treatment. Derm. Ther..

[138] Resende J.H.C., de Aquino G.S.T., do Nascimento F.R.F., Aguiar M.M., Fiorelli R.K.A. (2015). Oral Use of an Infusion of Leaves of *Solanum paniculatum* L., *Jacaranda brasiliensis* and *Sonchus oleraceus* for Treatment of Vitiligo. J. Cosmet. Dermatol. Sci. Appl..

